# Identification of beta-arrestin-1 as a diagnostic biomarker in lung cancer

**DOI:** 10.1038/s41416-018-0200-0

**Published:** 2018-08-06

**Authors:** Victoria El-Khoury, Mélanie Béland, Anna Schritz, Sang-Yoon Kim, Petr V. Nazarov, Louis Gaboury, Katriina Sertamo, François Bernardin, Roxane Batutu, Laurent Antunes, Catherine W. Bennett, François Faÿs, Guy Berchem, Yeoun Jin Kim

**Affiliations:** 1 Proteome and Genome Research Unit, Department of Oncology, Luxembourg Institute of Health, 1 A-B Rue Thomas Edison, L-1445 Strassen, Luxembourg; 2 Histology and Molecular Pathology Research Unit, Institute for Research in Immunology and Cancer (IRIC), Université de Montréal, 2950, chemin de Polytechnique, Pavillon Marcelle-Coutu, Montréal, QC H3T 1J4 Canada; 3 Competence Center for Methodology and Statistics, Luxembourg Institute of Health, 1 A-B Rue Thomas Edison, L-1445 Strassen, Luxembourg; 4 Department of Pathology and Cell Biology, Faculty of Medicine, Université de Montréal, CP 6128, Succursale Centre-ville, Montréal, QC H3C 3J7 Canada; 5 Clinical and Epidemiological Investigation Center, Luxembourg Institute of Health, 1 A-B Rue Thomas Edison, L-1445 Strassen, Luxembourg; 6 Integrated BioBank of Luxembourg, 1, rue Louis Rech, L-3555, 3531 Dudelange, Luxembourg; 7 Laboratory of Experimental Cancer Research, Department of Oncology, Luxembourg Institute of Health, 84, rue Val Fleuri, L-1526 Luxembourg, Luxembourg; 8 Centre Hospitalier de Luxembourg, 4 rue Nicolas-Ernest Barblé, L-1210 Luxembourg, Luxembourg; 9 Present Address: NantOmics, LLC, 9600 Medical Center Dr #300, Rockville, MD 20850 USA

**Keywords:** Non-small-cell lung cancer, Predictive markers, Diagnostic markers

## Abstract

**Background:**

Distinguishing lung adenocarcinoma (ADC) from squamous cell carcinoma (SCC) has a tremendous therapeutic implication. Sometimes, the commonly used immunohistochemistry (IHC) markers fail to discriminate between them, urging for the identification of new diagnostic biomarkers.

**Methods:**

We performed IHC on tissue microarrays from two cohorts of lung cancer patients to analyse the expression of beta-arrestin-1, beta-arrestin-2 and clinically used diagnostic markers in ADC and SCC samples. Logistic regression models were applied for tumour subtype prediction. Parallel reaction monitoring (PRM)-based mass spectrometry was used to quantify beta-arrestin-1 in plasma from cancer patients and healthy donors.

**Results:**

Beta-arrestin-1 expression was significantly higher in ADC versus SCC samples. Beta-arrestin-1 displayed high sensitivity, specificity and negative predictive value. Its usefulness in an IHC panel was also shown. Plasma beta-arrestin-1 levels were considerably higher in lung cancer patients than in healthy donors and were higher in patients who later experienced a progressive disease than in patients showing complete/partial response following EGFR inhibitor therapy.

**Conclusions:**

Our data identify beta-arrestin-1 as a diagnostic marker to differentiate ADC from SCC and indicate its potential as a plasma biomarker for non-invasive diagnosis of lung cancer. Its utility to predict response to EGFR inhibitors is yet to be confirmed.

## Introduction

Lung cancer is the deadliest cancer worldwide. It is commonly categorised into small cell lung cancer (SCLC, 15–20%) and non-small cell lung cancer (NSCLC, 80–85%).^1,2^ The rise of personalised medicine has been accompanied by an increasing need for a thorough classification of NSCLC. Indeed, targeted drugs approved for the treatment of specific NSCLC subtypes were either ineffective or harmful if used in other NSCLC groups.^3,4^ In this regard, distinguishing between adenocarcinoma (ADC) and squamous cell carcinoma (SCC) has a tremendous therapeutic implication.^5–9^

In the majority of cases, haematoxylin–eosin staining is sufficient to distinguish ADC from SCC. However, the discrimination between these subtypes is more challenging in poorly differentiated tumours or in small biopsies with few cancer cells. Therefore, according to the 2015 World Health Organization (WHO) Guidelines, immunohistochemistry (IHC) is sometimes mandatory for NSCLC subclassification.^10^ However, the routinely used IHC markers suffer from a suboptimal sensitivity and/or low specificity, thus underpinning the need for novel biomarkers to assist in accurate distinction between lung ADC and SCC.

The non-visual beta-arrestin-1 (also called arrestin-2 and encoded by the *ARRB1* gene) and beta-arrestin-2 (also called arrestin-3 and encoded by the *ARRB2* gene) belong to a family of four cytosolic adaptor proteins, known for their role in the desensitisation of the seven-transmembrane receptors.^11,12^ Beta-arrestins can also recruit cytoplasmic proteins and modulate downstream signalling pathways.^12–16^ Here, we describe the clinical potential of beta-arrestin-1 as a diagnostic marker to discriminate ADC from SCC, using tissue samples from independent patients’ cohorts. Additionally, we demonstrate the possible utility of beta-arrestin-1 as a plasma biomarker for non-invasive diagnosis of lung cancer and report preliminary results suggesting that beta-arrestin-1 could be useful to predict tumour’s response to epidermal growth factor receptor (EGFR) inhibitor therapy.

## Materials and methods

### Study subjects

Subjects in this study are either lung cancer patients followed at different hospitals in Luxembourg or healthy donors. Both groups signed an informed consent according to the Declaration of Helsinki. Two additional patient cohorts are related to the commercial tissue microarray (TMA) slides provided by amsbio and US Biomax (see below).

Lung cancer patients from the Luxembourg cohort donated tissue and/or blood. Healthy volunteers donated blood samples. Tissue samples (primary and/or metastatic) were obtained from 27 ADC and 11 SCC patients; their clinicopathological features are summarised in Supplementary Table 1. Except for patient no. 31 whose last anticancer treatment was 8 months before inclusion, all the other patients had never been treated with anticancer drugs at the time of tissue collection. Blood samples were obtained from 128 lung cancer patients (*n* = 72 ADC; *n* = 24 SCC; *n* = 32 other lung cancer subtypes, including adenosquamous carcinoma and NSCLC not otherwise specified (NOS)) and from 93 healthy donors. An overview of the clinicopathological features of patients and healthy donors whose blood samples were used in this study is provided in Supplementary Table 2. The overall survival (OS) was calculated from the date of sample collection to the date of death or last follow-up visit.

The study was approved by the national research ethics committee in Luxembourg “Comité National d’Ethique de Recherche” (CNER), and authorised by the national commission for data protection “Commission Nationale pour la Protection des Données” (CNPD). Diagnosis, staging and grading were done by two expert pathologists following the IASLC/ATS/ERS (International Association for the Study of Lung Cancer/American Thoracic Society/European Respiratory Society) classification of lung cancer (2011) and TNM (tumour, node and metastasis) classification of lung carcinoma (2009). All of the samples were processed following the standard operating procedures of the “Integrated Biobank of Luxembourg” (IBBL) to prepare formalin-fixed paraffin-embedded (FFPE) tissues and plasma samples.

Subjects in the amsbio cohort were ADC (*n* = 19) and SCC (*n* = 21) patients whose primary tumour tissues are included in the amsbio lung tumour tissue array (reference Z7020062). Samples with different or uncertain diagnosis and cases with non-representative cores or non-available IHC results were not considered. Supplementary Table 3 recapitulates the available clinicopathological features of the 40 selected patients in the amsbio cohort as provided by the manufacturer (the term “brionchioalveolar carcinoma” reported in this table was discontinued in the 2015 WHO classification of lung adenocarcinoma^10^).

Subjects in the US Biomax cohort were ADC (*n* = 27) and SCC (*n* = 44) patients whose primary tumour tissues are included in the US Biomax lung cancer microarray panel (reference LC20810). Samples with different diagnosis or extensive necrosis, and cases with non-representative cores or non-available IHC results were not considered. Supplementary Table 4 summarises the clinicopathological features of the 71 selected patients of the US Biomax cohort, as provided by the manufacturer.

### Construction of the in-house TMA

TMA construction was performed at the Institute for Research in Immunology and Cancer (IRIC) using tissue samples from the Luxembourg cohort and is herein referred to as in-house TMA. FFPE samples (tumour and, when available, distant “normal” tissues) were arrayed in duplicate using a tissue arrayer with 1 mm-diameter punches.

### Immunohistochemical analysis

Protein expression was assessed on 4 µm TMA sections using an automated IHC stainer and 3,3’-diaminobenzidine (DAB)-based visualisation. All of the sections were counterstained with haematoxylin. The antibodies against beta-arrestin-1 (ARRB1) (#30036), beta-arrestin-1-2 (ARRB1-2) (#4674) and beta-arrestin-2 (ARRB2) (#3857) were obtained from Cell Signaling Technology (Beverly, MA, USA). Anti-Thyroid Transcription Factor 1 (TTF1) (#M3575), anti-keratin 5-6 (KRT5-6) (#M7237) and anti-keratin 7 (KRT7) (#M7018) antibodies were obtained from Dako (Agilent Technologies, Mississauga, Ontario, Canada). Antibodies against Tumour Protein p63 (p63) (#CM163C) and Napsin A (NAPSA) (#NCL-L-Napsin A) were obtained from Biocare Medical (Pacheco, CA, USA) and Leica Biosystems (Concord, ON, Canada), respectively.

Quantitative image analysis was performed with the Visiomorph DP software (Visiopharm, Broomfield, CO, USA). The regions of interest (ROIs), composed of tumour cells, were chosen distant from necrotic areas. A Visiomorph score (VIS score) representing the mean intensity of the staining in each core was generated.

The positivity/negativity of each staining was evaluated by the pathologist according to the following criteria: a core was not representative if it contained less than 50 cancer cells. A case was considered positive if greater than 10% of cancer cells were stained. A staining was considered positive if it was nuclear (and, for p63, diffuse) for TTF1 and p63, cytoplasmic/membranous for KRT7 and KRT5, cytoplasmic and granular for NAPSA, and cytoplasmic/membranous (with or without nuclear staining) for beta-arrestin-1-2, beta-arrestin-1 and beta-arrestin-2. The positivity/negativity of the stains were used to calculate the sensitivity, specificity, positive predictive value (PPV) and negative predictive value (NPV) of each marker.

### Plasma processing

Plasma samples (40 µL) were processed to generate tryptic peptides as previously reported^17^ with a modification of the depletion step where 14 high-abundant plasma proteins were removed using MARS Hu-14 column (Agilent Technologies, Diegem, Belgium). Peptide mixtures (tryptic digest) were dried in vacuo after desalting with C18 SepPack column (Waters, Milford, MA). Samples were reconstituted with 200 µL of 0.1 % formic acid/4% acetonitrile, and further diluted with the internal standard to 4:1 (sample: internal standard) volume ratio. Then, 1 µL of the final sample was used for liquid chromatography-parallel reaction monitoring (LC-PRM) analysis.

### LC-PRM analysis

A high-purity tryptic peptide EDLDVLGLTFR, unique to beta-arrestin-1, was synthesised incorporating heavy isotope (^13^C_6_^15^N_4_) for the C-terminal Arg by Thermo Fisher Scientific (Rockford, IL, USA). A preliminary LC-mass spectrometry (MS) analysis was performed in order to determine the best precursor ion and retention time of the peptide. Final concentration of 40  fmol/µL of the peptide was spiked in all of the samples. LC-PRM analyses were performed on a Q-Exactive Plus mass spectrometer coupled with an Ultimate 3000 RSLCnano system (Thermo Fisher Scientific, Bremen, Germany) as previously described.^18^

For PRM-based quantification, product ions of light (endogenous) and heavy form of EDLDVLGLTFR peptide were extracted at the expected retention time with an *m/z* window of 1.5 min. The most intense production of the heavy peptide was used for quantification and the ratio of light/heavy peak area was used to calculate plasma concentration.

### Statistical analysis

The non-parametric Kruskal–Wallis test was used to compare different groups. Mann–Whitney rank sum test was applied for pairwise comparisons. One-way analysis of variance with Tukey's post-hoc analysis were used to compare The Cancer Genome Atlas (TCGA) data.

To determine whether tissue and plasma levels of beta-arrestin-1 and/or beta-arrestin-1-2 were associated with OS, Cox proportional hazards regression models were applied on log-transformed data and the analysis was done using the “Survival” package of R statistical software. The log-rank test was used to compare the survival distributions of the samples. *P* value < 0.05 was considered statistically significant.

Bootstrap sampling and least absolute shrinkage and selection operator (Lasso) penalisation were used on the in-house and the amsbio datasets to find a combination of proteins to use for the prediction of lung cancer subtype. Bootstrap was performed on 1000 samples drawn from each original dataset. Lasso penalisation in logistic regression models (with 10-fold cross-validation) were applied on each sample using the “glmnet” package of R. Proteins retained in more than 75% of the cases were selected as most predictive.

Logistic regression models using Firth’s penalised likelihood method were applied. Procedure PROC LOGISTIC with Firth penalisation of the statistical software SAS was used. Univariable models were run first in order to investigate each protein separately. Then, logistic regression models were run using the resulting variable combination of Lasso. All logistic regression models were run separately for each dataset. To evaluate the accuracy of the models in predicting lung cancer subtype, receiver operating characteristic (ROC) curves with their associated area under ROC curves (AUC) were computed separately for each. The Akaike information criterion (AIC) was calculated to estimate the quality of the model relative to other models.

## Results

### Expression patterns of beta-arrestin-1 and 2 and commonly used immunohistochemical markers in the in-house TMA

**Fig. 1:**
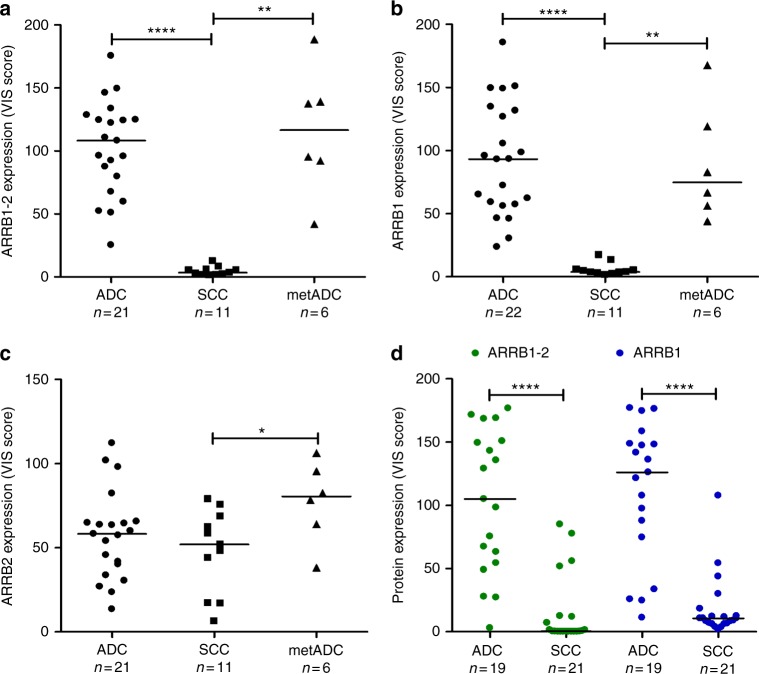
Beta-arrestin-1 and 2 protein expression in lung ADC and SCC samples in the in-house and the amsbio TMAs. Scatter plots represent quantitative analysis (VIS score) of IHC results in sections of **a**–**c** the in-house TMA and of **d** the amsbio TMA. Sections of the in-house TMA were incubated with an antibody that (**a**) recognises both beta-arrestin-1 and 2 (ARRB1-2), or specifically detects either (**b**) beta-arrestin-1 (ARRB1) or **c** beta-arrestin-2 (ARRB2). **d** Sections of the amsbio TMA were incubated with the anti-beta-arrestin-1-2 antibody or with the specific anti-beta-arrestin-1 antibody and “*n*” indicates the number of subjects in each group. Data points and their median are shown. ****P < 0.0001 **P < 0.01 and *P < 0.05 using Mann–Whitney rank sum test

**Fig. 2:**
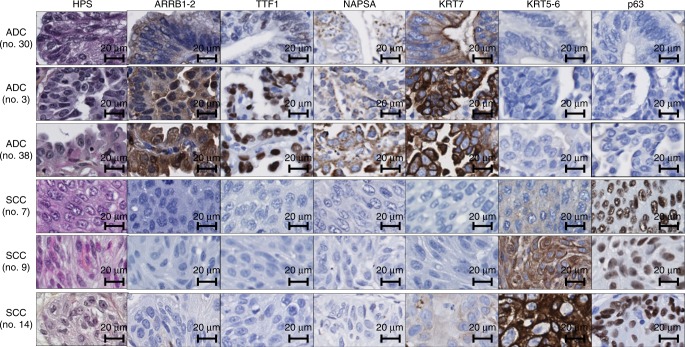
Representative images of HPS and IHC stainings in lung ADC and SCC samples. Automated IHC was performed on sections of the in-house TMA using antibodies that recognise beta-arrestin-1-2 (ARRB1-2), the commonly used ADC markers (TTF1, NAPSA, KRT7) and SCC markers (KRT5-6 and p63). DAB-based visualisation was used to assess protein expression. For each sample, a haematoxylin/phloxine/saffron (HPS)-stained slide is shown

To find new IHC markers distinguishing ADC from SCC, the expression of 77 potential biomarkers was analysed by IHC on in-house TMA sections. Interestingly, all ADC samples (primary and metastatic) expressed beta-arrestin-1-2, as opposed to the 11 SCC samples (results from one ADC sample were unavailable) (Fig. 1a). Representative IHC staining patterns are shown in Fig. 2. The haematoxylin/phloxine/saffron (HPS) stain illustrates a glandular differentiation and a lepidic growth pattern in ADC samples (patients 3 and 38, respectively), and keratin pearls and intercellular bridges in SCC samples (patients 9 and 7, respectively). IHC results are concordant with the diagnosis: ADC samples express TTF1, NAPSA and KRT7, whereas SCC samples express KRT5-6 and p63. The low specificity of KRT7 for ADC is illustrated by one example of weak positivity in SCC (patient 14). Beta-arrestin-1-2 showed positive staining in ADC but not in SCC samples.

**Fig. 3:**
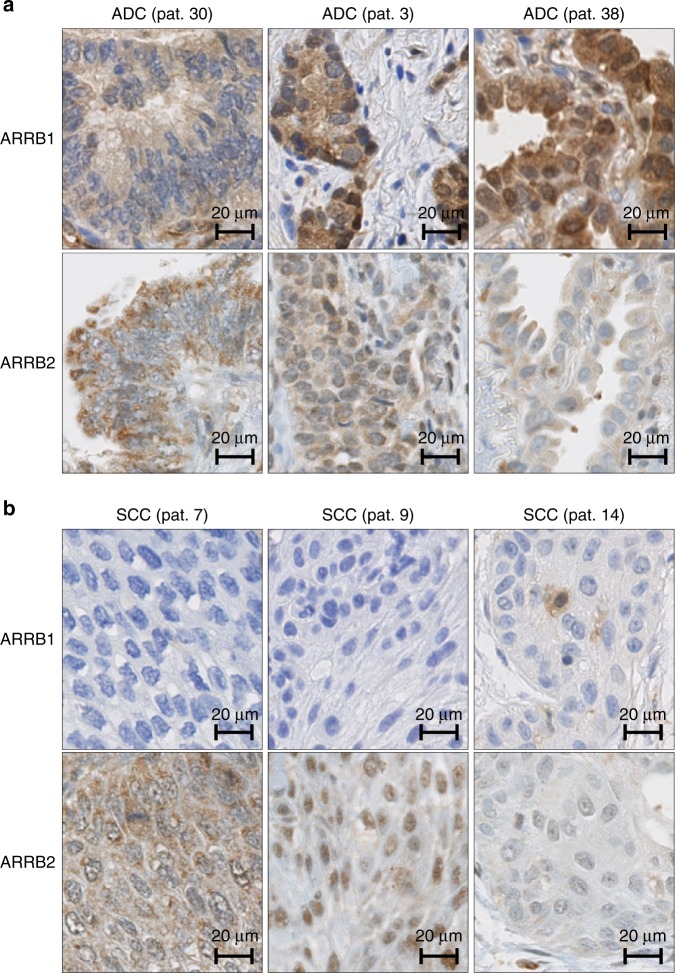
Representative images of beta-arrestin-1 (ARRB1) and beta-arrestin-2 (ARRB2) IHC staining in lung ADC and SCC samples. Automated IHC was performed on sections from the in-house TMA using antibodies that recognise specifically beta-arrestin-1 or beta-arrestin-2. DAB-based visualisation was used to assess protein expression. IHC images in **a** three ADC samples and **b** three SCC samples are shown. Results from the same patient samples as those represented in Fig. 2 are displayed here

To identify which beta-arrestin is responsible for this differential expression, we then performed IHC staining using specific anti-beta-arrestin-1 and anti-beta-arrestin-2 antibodies. As illustrated in Fig. 1b, c, beta-arrestin-1 expression was significantly higher in ADC versus SCC samples, whereas beta-arrestin-2 was expressed at similar levels in both primary NSCLC subtypes. Figure 3 shows the beta-arrestin-1 and beta-arrestin-2 stains corresponding to the samples described in Fig. 2. As illustrated, ADC cases were beta-arrestin-1 positive, whereas SCC samples were beta-arrestin-1 negative. Beta-arrestin-2 was expressed in all six samples at variable intensities. Interestingly, normal pneumocytes express beta-arrestin-1 protein (Supplementary Figure 1), thus raising the question of whether the overexpression of beta-arrestin-1 in ADC compared to SCC samples is due to a loss of its expression in the normal cells that evolved to SCC cells during the carcinogenesis process. The analysis of RNA-sequencing (RNA-seq) data downloaded from the TCGA data portal revealed that (1) *ARRB1* and *ARRB2* messenger RNAs (mRNAs) were significantly overexpressed in ADC compared to SCC samples with a higher difference of expression observed for *ARRB1* mRNA, and (2) the expression of *ARRB1* mRNA appeared considerably higher in normal cells from the non-tumoural lung tissue than in the SCC tissue, explaining in part the results obtained at the protein level (Supplementary Figure 2). Altogether, these data strongly suggest that beta-arrestin-1 may be a suitable marker for distinguishing ADC from SCC. Of note, we did not find any significant difference in OS of patients in the Luxembourg cohort related to beta-arrestin-1 or beta-arrestin-1-2 expression in cancer tissue (*P* value (log-rank test) = 0.37 and 0.59), respectively). Similarly, when considering only ADC patients, no association could be demonstrated between OS and beta-arrestin-1 or beta-arrestin-1-2 expression (*P* value (log-rank test) = 0.78 and 0.47).

The IHC results obtained in primary ADC and SCC samples of the in-house TMA and the diagnostic accuracy of the markers are summarised in Table 1. Interestingly, in ADC, sensitivity and specificity of beta-arrestin-1-2 were of 100%, outperforming all the canonical differential markers investigated. The only SCC sample that was beta-arrestin-1 positive contained necrotic regions (outside of the ROI delimited for quantification). Whereas the specificities of TTF1 and NAPSA were of 100%, their diagnostic sensitivities (73 and 70%, respectively) and their NPV (65% for TTF1 and NAPSA versus 100% for beta-arrestin-1-2 and beta-arrestin-1) were much lower. Despite its exceptional sensitivity, KRT7 displayed a low specificity (82%) for ADC. In SCC, KRT5-6 and p63 were equally sensitive (91%) and specific (100%).

**Table 1:** Diagnostic accuracy of the IHC markers in primary lung ADC and SCC in the in-house and the amsbio TMA panels

IHC markers	ARRB1-2		ARRB1		TTF1		NAPSA		KRT7		KRT5-6		p63	
Measures	In-house	Amsbio	In-house	Amsbio	In-house	Amsbio	In-house	Amsbio	In-house	Amsbio	In-house	Amsbio	In-house	Amsbio
Sensitivity (%)	100	95	100	95	73	68	70	89	100	95	91	90	91	90
Specificity (%)	100	81	91	81	100	100	100	100	82	90	100	100	100	100
Positive predictive value (PPV) (%)	100	82	96	82	100	100	100	100	91	90	100	100	100	100
Negative predictive value (NPV) (%)	100	94	100	94	65	78	65	91	100	95	96	90	96	90

ARRB1-2, ARRB1, TTF1, NAPSA and KRT7 were evaluated according to their performance in staining ADC. KRT5-6 and p63 were evaluated according to their performance in staining SCC. Data are shown as percentages

Importantly, the poor sensitivity of TTF1 in primary ADC seemed to be linked to tumour grade (Supplementary Table 5). Indeed 87.5% of well-differentiated ADC samples (7/8) and only 60% of moderately to poorly and poorly differentiated ADC (3/5) were TTF1 positive. Similarly, NAPSA had a better sensitivity in well-differentiated ADC (6/7) versus moderately and poorly differentiated ADC (5/9). Interestingly, anti-beta-arrestin-1-2 and anti-beta-arrestin-1 stained ADC irrespective of tumour grade. Additionally, anti-beta-arrestin-1 stained positive for all 6 metastatic lung adenocarcinoma in specimens from their metastatic sites, whereas anti-TTF1 and anti-NAPSA unambiguously stained only 2 (from patients 19 and 29) and 3 samples (from patients 22, 19 and 34), respectively (Supplementary Figure 3). These findings suggest that beta-arrestin-1 might be of particular utility when a diagnosis has to be made in poorly differentiated primary NSCLC or in metastatic samples of NSCLC.

### Data validation using commercially available TMAs

To validate the differential diagnostic ability of beta-arrestin-1 in an independent cohort, we performed IHC staining using a lung tumour TMA from amsbio. Similar to the data obtained with the in-house TMA, beta-arrestin-1 (detected by either anti-beta-arrestin-1 or anti-beta-arrestin-1-2 antibodies) was significantly downregulated in SCC (*n* = 21) versus ADC (*n* = 19) (Fig. 1d). Ninety-five percent of ADC samples (18/19) were beta-arrestin-1-2 positive and the same percentage of beta-arrestin-1-positive ADC cases was obtained. Seventeen SCC samples were beta-arrestin-1-2- and beta-arrestin-1 negative, whereas 4 SCC samples were positive for both. These findings confirm the high sensitivity of beta-arrestin-1-2 and beta-arrestin-1 in staining ADC (95%), which is comparable to the sensitivity of KRT7 (95%) and higher than that of TTF1 (68%) and NAPSA (89%) (Table 1). In addition, the results from the amsbio panel emphasise the high NPV of beta-arrestin-1 and beta-arrestin-1-2 (94%), surpassing the NPV of TTF1 (78%) and NAPSA (91%). Similar to the in-house TMA data, TTF1 and NAPSA demonstrated 100% specificity. Nevertheless, the amsbio TMA data showed poor beta-arrestin-1-2 and beta-arrestin-1 specificities (81%) when compared to their performance in the in-house TMA or when compared to other ADC markers in the amsbio panel. The reasons behind these discrepancies are unknown; however, differences in treatment status at sample collection time between both cohorts may partially explain these inconsistencies.

Here, it was not possible to link the differentiation of the tumour with the sensitivity of ADC markers given that there were no well-differentiated ADC and only 2 poorly differentiated ADC samples among the samples with available grading information. Nevertheless, the results obtained in poorly differentiated tumours contrasted with the data obtained in the in-house TMA (4/9 SCC samples were beta-arrestin-1-2- and beta-arrestin-1-positive and one ADC sample was beta-arrestin-1-negative) (Supplementary Table 6). These discrepancies may be due to the fact that the grading information provided by amsbio was obtained from different pathologists across several hospitals, and that interpretation of histology specimens is prone to subjectivity. Further investigation is needed to determine whether beta-arrestin-1 may be of interest in the diagnosis of poorly differentiated ADC.

The beta-arrestin-1-2 staining was also evaluated in ADC and SCC samples from the US Biomax lung TMA panel. As shown in Supplementary Figure 4, beta-arrestin-1-2 expression was significantly higher in ADC samples (*n* = 27) compared to SCC samples (*n* = 44), confirming our previous findings. Unfortunately, a lot of tissue cores included in the US Biomax panel were moderately to extremely necrotic, thus precluding any further analysis.

Altogether, these data highlight the potential of beta-arrestin-1 as a differential diagnostic marker to discriminate ADC from SCC.

Next, we sought to determine whether beta-arrestin-1 as a single marker or in a panel of IHC markers can improve the differentiation between ADC and SCC.

### Determination of the relevant markers to combine for tumour subtype prediction

We first determined the most predictive variables to combine for predicting tumour subtype (ADC or SCC) based on the IHC scores. To do so, bootstrap sampling and Lasso penalisation were conducted separately on data from each TMA panel. For the in-house TMA dataset, the proteins that showed high ability of prediction were beta-arrestin-1-2, KRT7, KRT5-6 and p63. For amsbio TMA dataset, KRT7 and KRT5-6 were identified as the most predictive.

### Application of univariable and multivariable models for tumour subtype prediction

To analyse the predictive performance of the proteins, in-house and amsbio TMA datasets were used separately to derive univariable and multivariable logistic regression models. For multivariable models, beta-arrestin-1-2, KRT7, KRT5-6 and p63, selected as most predictive by Lasso, were used. The models were then applied to both datasets for predicting tumour subtype.

In the upper part of Table 2, logistic regression models were performed on in-house TMA values. Using the estimated parameters of each model, predictions for cancer subtype were done on the in-house and amsbio TMA datasets. Generally speaking, the in-house dataset was used as the training dataset and the amsbio TMA values were used as the test dataset. To evaluate the strength of prediction, AUCs were calculated. The AIC is also shown in order to estimate how well the model fits the data. In the lower part of Table 2, the same procedure was done using the amsbio TMA dataset as the training and the in-house TMA dataset as the test datasets. Among the univariable models derived from the in-house TMA, those which included beta-arrestin-1-2 and beta-arrestin-1 displayed the lowest AIC and the highest AUC for predictions on the in-house TMA dataset. However, when predicting subtype of cancer in amsbio TMA dataset, models with beta-arrestin-1-2 and beta-arrestin-1 did not perform as well (AUC = 0.930 and 0.945). Univariable models with KRT7, KRT5-6 or p63 showed higher AUC when prediction was done on amsbio dataset (AUC = 0.977, 0.972 and 0.972 for KRT7, KRT5-6 and p63, respectively). Similarly, when models were performed on the amsbio TMA dataset, beta-arrestin-1-2 and beta-arrestin-1 had the highest AUC when predicting cancer subtype in the in-house TMA (AUC = 1.000 for beta-arrestin-1-2 and beta-arrestin-1). In contrast, when predicting in amsbio TMA, KRT7 showed the highest AUC value (AUC = 0.976) compared to all other univariable models. Among all of the univariable and multivariable models, and in both datasets, the model that best fitted the data was the 4-protein combination beta-arrestin-1-2+KRT7+KRT5-6+p63 (AIC = −14.069 and −12.370 in the in-house and amsbio TMA datasets, respectively). Moreover, this model displayed high AUC values when the results of the training datasets were used to predict subtype of cancer on the test dataset (AUC = 0.998 and 1.000). In addition to this 4-protein combination, other combinations seemed to have a good performance, including the 3-protein combinations KRT7+KRT5-6+p63 (AIC = −7.583 and AUC = 1.000 in amsbio test dataset (Table 2 upper part) and AIC = −5.005 and AUC = 1.000 in the in-house test dataset (Table 2 lower part)) and beta-arrestin-1-2+KRT7+KRT5-6 (AIC = −9.216 and AUC = 0.998 in amsbio test dataset (Table 2 upper part), and AIC = −7.006 and AUC = 1.000 in the in-house test dataset (Table 2 lower part)). In conclusion, according to the results of Lasso, the AIC and AUC values, the combination of beta-arrestin-1-2+KRT7+KRT5-6+p63 seems to provide the best differentiation of ADC from SCC.

**Table 2:** Performance of the logistic regression models derived using the in-house and the amsbio TMA datasets

		Prediction on training dataset	Prediction on test dataset
Models derived using the in-house TMA dataset as training dataset			
Univariable models			
Parameters	AIC	AUC	AUC
ARRB1-2	2.949	1.000	0.930
ARRB1	3.151	1.000	0.945
KRT7	3.518	0.995	0.977
KRT5-6	7.842	0.909	0.972
p63	6.212	0.995	0.972
TTF1	15.750	0.967	0.877
NAPSA	14.995	0.967	0.865
Multivariable models			
ARRB1-2+KRT7+KRT5-6+p63	−14.069	1.000	0.998
KRT7+KRT5-6+p63	−7.583	1.000	1.000
KRT5-6+KRT7	−1.686	1.000	1.000
ARRB1-2+KRT5-6+p63	−7.409	1.000	0.990
ARRB1-2+KRT7+p63	−7.074	1.000	0.998
ARRB1-2+KRT7+KRT5-6	−9.216	1.000	0.998
ARRB1-2+KRT5-6	−1.704	1.000	0.990
ARRB1-2+KRT7	−0.576	1.000	0.980
ARRB1-2+p63	0.107	1.000	0.988
KRT5-6+p63	0.104	1.000	0.998
KRT7+p63	−0.078	1.000	0.995
Models derived using the amsbio TMA dataset as training dataset			
Univariable models			
Parameters	AIC	AUC	AUC
ARRB1-2	21.611	0.931	1.000
ARRB1	20.135	0.944	1.000
KRT7	12.078	0.976	0.996
KRT5-6	9.254	0.971	0.909
p63	14.293	0.971	0.996
TTF1	27.401	0.870	0.968
NAPSA	27.751	0.865	0.967
Multivariable models			
ARRB1-2+KRT7+KRT5-6+p63	−12.370	0.998	1.000
KRT7+KRT5-6+p63	−5.005	1.000	1.000
KRT5-6+KRT7	0.408	1.000	1.000
ARRB1-2+KRT5-6+p63	−2.542	0.990	1.000
ARRB1-2+KRT7+p63	−2.458	0.995	1.000
ARRB1-2+KRT7+KRT5-6	−7.006	0.998	1.000
ARRB1-2+KRT5-6	3.868	0.990	1.000
ARRB1-2+KRT7	5.674	0.977	1.000
ARRB1-2+p63	4.646	0.990	1.000
KRT5-6+p63	3.809	0.995	1.000
KRT7+p63	5.771	0.990	1.000

### Identification of beta-arrestin-1 as a potential plasma biomarker for non-invasive diagnosis of lung cancer and prediction of tumour response to EGFR inhibitors

**Fig. 4:**
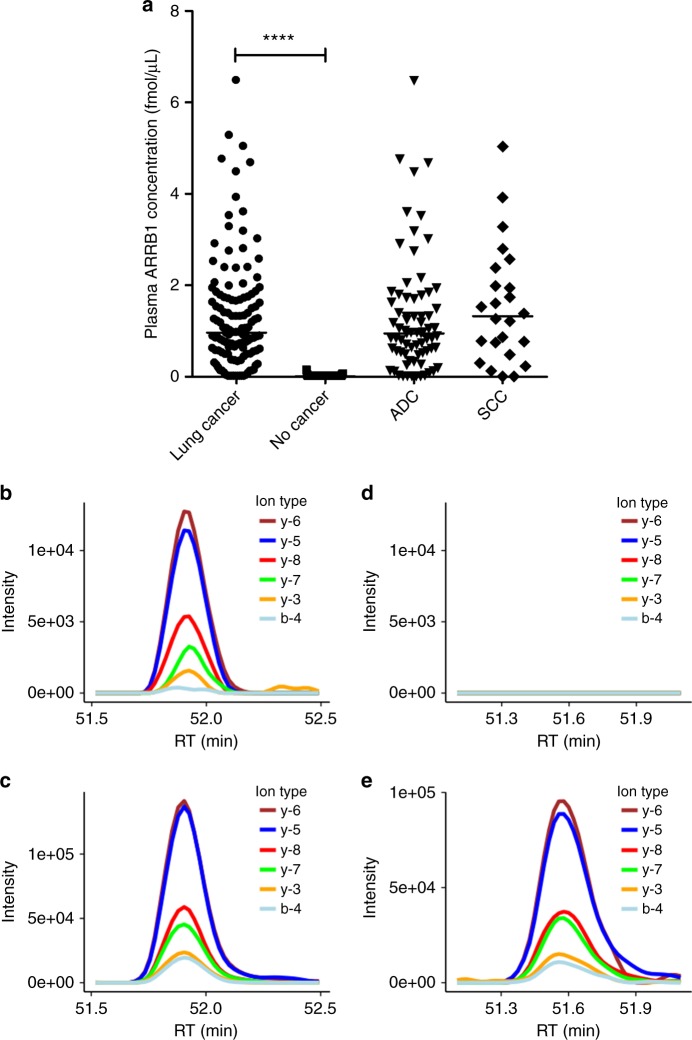
Plasma levels of beta-arrestin-1 (ARRB1) in lung cancer patients and healthy donors. **a** Scatter plots of plasma beta-arrestin-1 concentration obtained from lung cancer patients (*n* = 128) and healthy volunteers (*n* = 93) using the PRM assay targeting EDLDVLGLTFR. Data points and their median are shown. PRM data from the lung cancer patients were further divided into ADC (*n* = 72) and SCC (*n* = 24) groups in the same graph. PRM readout of beta-arrestin-1 measured in one of the lung cancer patient samples is shown in **b** with the traces of detected product ions of the peptide EDLDVLGLTFR compared to the ones of internal standard peptide shown in **c**. PRM readout of beta-arrestin-1 measured in one of the healthy volunteer samples is shown in **d** indicating no signal was detected compared to the internal standard shown in **e**. ****P < 0.0001 using Mann–Whitney rank sum test

Next, we sought to investigate whether circulating beta-arrestin-1 could aid in the non-invasive diagnosis of lung cancer. PRM-based MS was used to quantify beta-arrestin-1 in plasma from 128 lung cancer patients and 93 healthy donors. Although beta-arrestin-1 levels were comparable in ADC and SCC samples, beta-arrestin-1 concentrations were significantly higher in plasma from lung cancer patients when compared to healthy donors (Fig. 4a). Examples of the PRM readouts for beta-arrestin-1-specific peptide compared to its heavy-isotope-labelled internal standard peptide in a lung cancer sample (Fig. 4b, c) and in a non-cancer sample (Fig. 4d, e) are shown. The plasma levels of beta-arrestin-1 were not dependent on tumour stage, grade or lung cancer patients’ smoking habit. The reason behind these distinct beta-arrestin-1 plasma concentration profiles is still unknown. Additionally, the OS analysis did not reveal any significant prognostic value of plasma beta-arrestin-1 levels, neither in lung cancer patients in general (*P* value (log-rank test) = 0.67) nor in lung ADC patients (*P* value (log-rank test) = 0.47) nor in lung SCC patients (*P* value (log-rank test) = 0.08). In conclusion, these results indicate that beta-arrestin-1 is a potential circulating diagnostic marker for lung cancer.

Finally, we wondered whether quantification of beta-arrestin-1 in plasma may help in determining patients who would most likely respond to classical chemotherapy regimens or to EGFR inhibitor therapy (independently of whether treatments were administered as first-line therapy or beyond). Therefore, we analysed the expression of plasma beta-arrestin-1 (at baseline, i.e., before treatment) according to the best tumour response obtained after treatment. As shown in Supplementary Figure 5A, plasma beta-arrestin-1 levels could not predict response to classical chemotherapy, as no significant difference was detected among all 4 response groups (complete response (CR), *n* = 8; partial response (PR), *n* = 28; stable disease (SD), *n* = 16 and progressive disease (PD), *n* = 24). However, when analysing beta-arrestin-1 plasma levels according to tumour response to EGFR inhibitors (here CR and PR were combined in one group, since only 2 patients achieved CR; *n* = 11), we observed a trend towards increased beta-arrestin-1 baseline levels with worsening responses (a significant difference between patients with PD (*n* = 14) after EGFR inhibitor treatment and patients in the CR+PR group was shown (*P* value = 0.0173)) (Supplementary Figure 5B). Our findings open a new hypothesis that low plasma beta-arrestin-1 concentrations in lung cancer patients might predict favourable responses to subsequent EGFR inhibitor therapy. On the contrary, high levels of plasma beta-arrestin-1 would identify patients who are at risk to develop disease progression after EGFR inhibitor therapy. These results should be considered with caution due to the limited number of samples analysed. A larger patient cohort is needed to validate them.

## Discussion

The recent advances in personalised medicine have resulted in an increasing need for more accurate NSCLC subtyping.^8,19,20^ Here, we provided evidence that beta-arrestin-1 can effectively differentiate between lung ADC and lung SCC, and demonstrated its usefulness as a diagnostic marker in an IHC panel. Our data clearly show that the sensitivity of beta-arrestin-1 was either equal to or surpassed the ADC IHC markers commonly used in clinics. Although beta-arrestin-1 displayed a suboptimal specificity in the amsbio TMA panel, the IHC data demonstrated its exceptional specificity in the in-house TMA. The minor discrepancies observed between both TMA results may stem, at least in part, from the variable treatment status of the patients at the time of sample collection. Importantly, beta-arrestin-1 exhibited a very good NPV in both TMA panels. Additionally, we showed that beta-arrestin-1 plasma levels of lung cancer patients were considerably higher than those of healthy donors, implying its utility as a circulating diagnostic marker of lung cancer. Finally, we found that high plasma beta-arrestin-1 levels were observed in patients who would develop a progressive disease following EGFR inhibitor therapy. This finding sheds light on the potential that plasma beta-arrestin-1 might bear as a predictive biomarker of responsiveness to EGFR inhibitors and warrants additional efforts to validate it in independent studies.

Consistent with other studies, our results emphasise the high sensitivity and low specificity of KRT7 for ADC, and confirm the high specificity of TTF1 and NAPSA but their suboptimal sensitivity for ADC, mainly in poorly differentiated tumours.^8,21,22^ Interestingly, beta-arrestin-1 appeared highly sensitive and specific for ADC in the in-house TMA even in poorly differentiated cases. Moreover, beta-arrestin-1 outperformed TTF1 and NAPSA in the staining of the 6 lung ADC samples that metastasised to the soft tissue, bones, brain or lymph nodes. We do not claim that the ubiquitous beta-arrestin-1 can replace the relatively lung-specific TTF1 or NAPSA^21,23,24^ for the identification of lung as the primary site of cancer. However, it might help determine whether a lung cancer metastasis is an ADC or a SCC when no primary lung cancer tissue is available. The sensitivity and specificity of KRT5-6 and p63 for SCC obtained here are concordant with or exceed the sensitivity and specificity reported earlier.^8,21,25^

In accordance with our results, Dasgupta et al.^26^ did not find a substantially higher beta-arrestin-1 expression in primary ADC tissue when compared to normal lung tissue. However, the authors described increased beta-arrestin-1 levels in primary SCC relative to distant normal lung tissues, which is at odds with our findings. Our data showed similar beta-arrestin-1 expression in “normal” pneumocytes from ADC and SCC patients. If we consider that lung SCC cells derive from normal cells that evolved to a neoplastic state, then this observation would suggest that normal lung cells lose beta-arrestin-1 expression during the oncogenesis process leading to SCC. However, “normal” pneumocytes are probably not the best control cells for SCC. Rather, basal cells of the tracheobronchial compartment, thought to generate lung SCC, should be considered as normal control cells.^27,28^ In practice this constraint is challenging to overcome due to the unavailability or scarcity of these cells in the FFPE tissues. Keeping this caveat in mind, our data suggest that beta-arrestin-1 would be downregulated during the squamous differentiation process and/or the mechanism of oncogenesis in SCC, similar to the loss-of-function mutations in NOTCH1 and the amplification of SOX2 and TP63.^29,30^

*ARRB1* gene is located on the chromosomal band 11q13 and is altered in 5.2% of lung ADC cases and in 1.7% of lung SCC (information from the cBioPortal for cancer genomics).^31,32^ However, the prevalence of these alterations cannot explain the observed differences in beta-arrestin-1 expression. Interestingly, the *CCND1* gene maps to the same chromosomal region and appears significantly overexpressed in the ADC versus SCC samples of the in-house TMA (data unshown), in accordance with previous reports.^33,34^ Hence, the expression of genes harboured in the 11q13 region might be regulated by the same epigenetic events occurring at this locus and resulting in the co-expression/repression of neighbouring genes.

Given their diverse roles and ubiquitous expression, it is not surprising that beta-arrestins are involved in the regulation of multiple physiological processes including proliferation, differentiation and apoptosis^12,14,35^ and in tumour development and progression.^15,36^ The activation of the EP4 receptor/beta-arrestin-1/c-Src signalling was suggested to promote lung ADC cell migration.^37^ Similar findings were reported in colorectal cancer cells.^38^ Interestingly, beta-arrestin-1 was required for nicotine-mediated activation of c-Src and downstream signalling pathways leading to growth, progression, invasion and metastasis of NSCLC.^26,39,40^ In A549 cells, nicotine induced the expression of the mesenchymal genes, vimentin and fibronectin, in a beta-arrestin-1-dependent manner. However, it did not alter the expression of beta-arrestin-1 at the transcriptional or at the translational levels.^39^ This finding is in accordance with the absence of significant association between tumour beta-arrestin-1 expression (at the mRNA or protein levels) and smoking status of lung cancer patients in different studies.^39,41,42^ Instead, beta-arrestin-1 translocated to the nucleus upon nicotine stimulation where it bound the transcription factor E2F1 and this translocation was necessary for the induction of the epithelial*–*mesenchymal transition genes in A549 cells.^26,39^ These beta-arrestin-1-dependent nicotine effects were demonstrated only in ADC cells and have not been investigated in SCC. Since the risk of developing SCC is strongly associated with cigarette smoking^43^ and given that beta-arrestin-1 expression is considerably downregulated in SCC (as we demonstrated in this study), our data imply that the development and/or progression of nicotine-induced SCC would be at least partially controlled by beta-arrestin-1-independent mechanisms.

Qiu et al.^41^ claimed that high beta-arrestin-1 expression predicted poor prognosis in lung ADC. The same group made different statements in a subsequent paper, suggesting that loss of beta-arrestin-1 in both ADC and SCC was a predictor of poor survival, and that OS of ADC patients who showed beta-arrestin-1 expression in their cancer tissues was independent of its expression level.^44^ Our data do not support either of these statements: we did not find any link between beta-arrestin-1 levels (neither in cancer tissue nor in plasma) and patient OS.

Finally, we provided quantitative results implying the value of beta-arrestin-1 as a putative lung cancer diagnostic biomarker in plasma. A highly sensitive PRM method was used to measure the concentration of beta-arrestin-1 in plasma from lung cancer patients and healthy donors. The significant increase of plasma concentrations of beta-arrestin-1 specific to lung cancer samples is a promising novel finding that warrants further efforts to validate beta-arrestin-1 measurement in plasma as a powerful tool for non-invasive lung cancer diagnosis. In particular, more detailed investigations are required to reveal the context in which such measurement can assist physicians in diagnosing lung cancer. In addition to its putative value as non-invasive diagnostic tool, we hypothesised that plasma beta-arrestin-1 measurement might aid in the prediction of tumour response to EGFR inhibitor therapy. Indeed, we detected higher beta-arrestin-1 plasma concentrations in lung cancer patients who later developed disease progression after EGFR inhibitor treatment. This interesting finding requires detailed investigations before an affirmative conclusion can be drawn. Of note, beta-arrestin-1 is a cytosolic protein that translocates to the plasma membrane or to the nucleus; it is not described as a secreted protein and thus its detection in the plasma was not anticipated. Nevertheless, beta-arrestin-1 protein was previously identified in extracellular vesicles derived from various cancer cell lines^45^ and from primary monocyte-derived dendritic cells.^46^ Additionally, beta-arrestin-1 was detected in extracellular vesicles isolated from human milk^47^ and urine.^48^ These data suggest that beta-arrestin-1 may be shed by the cells and released into biofluids (in this case, plasma) via exosomes, microvesicles or other extracellular vesicles. These plasma vesicles may originate from cancer cells, from cells in the tumour microenvironment or from other cells in the body in response to the presence of the tumour.^49^ Importantly, exosomes derive from intracellular endosomal compartments^50^ and some receptor-beta-arrestin-1 complexes co-localise in endosomes,^51^ thus supporting the fact that beta-arrestin-1 may be part of the exosomal proteome. Finally, tissue leakage may result in beta-arrestin-1 release into the blood, thus contributing to the plasma pool of beta-arrestin-1.^1,52^

In conclusion, our data demonstrate the clinical potential of beta-arrestin-1 as a differential diagnostic marker in lung cancer and highlight the additional utility that it can bear as a non-invasive biomarker for the diagnosis and for prediction of response to EGFR inhibitor therapy in lung cancer.

## Electronic supplementary material

Supp table 1 - Clinicopathological features of lung cancer patients from the Luxembourg cohort who provided tissue samples

Supp table 2 - Clinicopathological features of lung cancer patients and healthy donors from the Luxembourg cohort who provided blood samples

Supp table 3 - Clinicopathological features of selected lung cancer patients from the amsbio cohort, as provided by the manufacturer

Supp table 4 - Clinicopathological features of selected lung cancer patients from the US Biomax cohort, as provided by the manufacturer

Supp table 5 - Positivity of IHC staining in primary lung tumours in the in-house TMA according to tumour grade

Supp table 6 - Positivity of IHC staining in primary lung tumours in the amsbio TMA according to tumour grade

Supp figure 1 - Beta-arrestin-1 (ARRB1) protein expression in lung ADC and SCC tissues and their distant « normal » counterparts

Supp figure 2 - Expression of ARRB1 and ARRB2 transcripts in lung ADC, lung SCC and non-tumour samples

Supp figure 3 - Representative images of HPS and IHC stainings in the metastatic sites of lung ADC

Supp figure 4 - Beta-arrestin-1-2 (ARRB1-2) protein expression in lung ADC and SCC samples in the US Biomax TMA

Supp figure 5 - Baseline plasma levels of beta-arrestin-1 (ARRB1) protein in lung cancer patients who subsequently experienced different tumour responses to chemotherapy and EGFR inhibitors

## Availability of data and material

The RNA-seq data were downloaded from the TCGA data portal.
