# Supplementary material for: Identification of beta-arrestin-1 as a diagnostic biomarker in lung cancer
Source: Br J Cancer. 2018 Aug 6;119(5):580–90. doi: 10.1038/s41416-018-0200-0 (PMC6162208; doi:10.1038/s41416-018-0200-0)
Supplement: Supplementary file 1 — Supp table 1 - Clinicopathological features of lung cancer patients from the Luxembourg cohort who provided tissue samples [file 41416_2018_200_MOESM1_ESM.pdf]

Supplementary Table 1. Clinicopathological features of lung cancer patients from the Luxembourg cohort who provided tissue samples.

Patient n°	Gender	Age at collection (y)	Smoking History (pack-year)	Diagnosis	Stage	Grade	Anatomic site of the collected tumour sample
1	M	70	Current smoker	SCC	IA	I	Lung
2	M	59	Current smoker	ADC	IA	N/A	Lung
3	F	50	Current smoker	ADC	IA	II	Lung
4	F	65	Former smoker	ADC	IB	N/A	Lung
5	M	77	Former smoker	SCC	IA	I	Lung
6	M	64	Never smoker	ADC	IIA	N/A	Lung
7	M	59	Former smoker	SCC	IIIA	I	Lung
8	F	64	Current smoker	ADC	IA	I	Lung
9	M	63	Current smoker	SCC	IA	I	Lung
10	F	85	Never smoker	ADC	IIB	I	Lung
11	M	75	Former smoker	ADC	IIIA	II	Lung
12	M	62	Current smoker	ADC	IB	I	Lung
13	F	55	Former smoker	ADC	IA	I	Lung
14	M	76	Former smoker	SCC	IA	I	Lung
15	M	69	Former smoker	SCC	N/A	I	Lung
16	M	62	Former smoker	ADC	IIB	III	Lung
17	F	72	Current smoker	ADC	N/A	III	Lung
18	M	59	Former smoker	SCC	IIIA	I	Lung
19	M	50	Never smoker	ADC	IV	I	Lung + soft tissue (metastasis)
20	F	65	Never smoker	ADC	IV	III	Lung
21	M	79	Former smoker	SCC	IIB	I	Lung
22	M	47	Former smoker	ADC	IV	I	Bone (metastasis)
23	M	73	Current smoker	ADC	IA	I	Lung
24	F	66	Former smoker	SCC	IA	III	Lung
25	M	49	Former smoker	ADC	IV	III	Bone (metastasis)
26	M	53	Never smoker	ADC	IV	N/A	Lymph node (metastasis)
27	M	74	Former smoker	ADC	IB	II~III	Lung
28	M	72	Former smoker	ADC	IA	III	Lung
29	F	54	Former smoker	ADC	IV	III	Brain (metastasis)
30	M	62	Former smoker	ADC	N/A	II	Lung
31	F	71	Never smoker	ADC	IV	N/A	Lung
32	F	76	Current smoker	ADC	IB	II	Lung
33	M	79	Former smoker	SCC	IIB	I	Lung
34	F	55	Former smoker	ADC	IV	III	Brain (metastasis)
35	M	67	Current smoker	ADC	IB	I	Lung
36	M	71	Former smoker	ADC	IA	II	Lung
37	M	83	Former smoker	SCC	IIA	III	Lung
38	F	84	Never smoker	ADC	IA	I	Lung

Diagnosis and staging of lung cancer patients were done by pathologists in Luxembourg's hospitals following the IASLC/ATS/ERS histological classification of lung tumours (2011) and TNM classification of lung carcinoma (2009). Grade I = well-differentiated; Grade II = moderately-differentiated; Grade III = poorly-differentiated.
N/A = information not available.
