# Supplementary material for: Identification of beta-arrestin-1 as a diagnostic biomarker in lung cancer
Source: Br J Cancer. 2018 Aug 6;119(5):580–90. doi: 10.1038/s41416-018-0200-0 (PMC6162208; doi:10.1038/s41416-018-0200-0)
Supplement: Supplementary file 2 — Supp table 2 - Clinicopathological features of lung cancer patients and healthy donors from the Luxembourg cohort who provided blood samples [file 41416_2018_200_MOESM2_ESM.pdf]

Supplementary Table 2. Clinicopathological features of lung cancer patients and healthy donors from the Luxembourg cohort who provided blood samples.

**Features**		**Lung cancer diagnosis**			**No cancer diagnosis**
		ADC	SCC	Others*	
N° of subjects		72	24	32	93
Gender	Female	34	4	13	42
	Male	38	20	19	51
Age at blood collection (y) [median value (range)]		63 (28-85)	71.5 (59-86)	62.5 (41-77)	62 (32-85)
Smoking history	Never smokers	14	0	1	14
	Former smokers	37	19	12	54
	Current smokers	21	5	6	25
Stage	I	13	8		
	II	3	5		
	III	4	5		
	IV	45	3		
	not available	7	3		
Grade	I	11	11		
	II	22	2		
	II~III	3	0		
	III	15	8		
	not available	21	3		
Anticancer treatment status before blood collection	Untreated	51	22	26	
	Treated	21	2	6	

*Other lung cancer types and subtypes including NSCLC NOS and adenosquamous carcinoma.

Diagnosis and staging of lung cancer patients were done by pathologists in Luxembourg's hospitals following the IASLC/ATS/ERS histological classification of lung tumours (2011) and TNM classification of lung carcinoma (2009). Grade I = well-differentiated; Grade II = moderately-differentiated; Grade III = poorly-differentiated.
