# Supplementary material for: Identification of beta-arrestin-1 as a diagnostic biomarker in lung cancer
Source: Br J Cancer. 2018 Aug 6;119(5):580–90. doi: 10.1038/s41416-018-0200-0 (PMC6162208; doi:10.1038/s41416-018-0200-0)
Supplement: Supplementary file 3 — Supp table 3 - Clinicopathological features of selected lung cancer patients from the amsbio cohort, as provided by the manufacturer [file 41416_2018_200_MOESM3_ESM.pdf]

Supplementary Table 3. Clinicopathological features of selected lung cancer patients from the amsbio cohort, as provided by the manufacturer.

Patient n°	Gender	Age	Diagnosis	TNM classification	Stage	Grade	Anatomic Site of the collected tumour sample
1	M	69	SCC	T2N0M0	IB	III	Lung
2	M	67	SCC	T3N0M0	IIB	III	Lung
3	M	60	SCC	T2N1M0	IIB	I~II	Lung
4	M	70	Bronchioloalveolar carcinoma	T2N1M0	IIB	II	Lung
5	M	59	ADC	T2N0M0	IB	II	Lung
6	M	49	ADC	T2N1M0	IIB	II~III	Lung
7	F	47	ADC	T2N0M0	IB	I~II	Lung
8	M	51	SCC	T2N1M0	IIB	I	Lung
9	M	45	Bronchioloalveolar carcinoma	T2N0M0	IB	N/A	Lung
10	F	55	ADC	T2N0M0	IB	III	Lung
11	M	43	SCC	T2N1M0	IIB	III	Lung
12	M	77	SCC	T2N0M0	IB	I~II	Lung
13	M	53	SCC	T2N0M0	IB	I~II	Lung
14	M	55	SCC	T2N1M0	IIB	III	Lung
15	F	42	ADC	T3N1M0	IIIA	II	Lung
16	M	74	ADC	T3N2M0	IIIA	II	Lung
17	M	48	ADC	T2N0M0	IB	II~III	Lung
18	F	39	SCC	T2N0M0	IB	III	Lung
19	M	54	SCC	T2N0M0	IB	III	Lung
20	F	66	Papillary ADC	T2N0M0	IB	N/A	Lung
21	M	55	ADC	T2N0M0	IB	N/A	Lung
22	M	55	SCC	T2N0M0	IB	II	Lung
23	M	53	ADC	T2N0M0	IB	III	Lung
24	M	57	SCC	T2N0M0	IB	II~III	Lung
25	M	44	SCC	T2N0M0	IB	II~III	Lung
26	M	53	SCC	T2N0M0	IB	III	Lung
27	M	65	SCC	T2N0M0	IB	I	Lung
28	M	47	Papillary ADC	T2N0M0	IB	N/A	Lung
29	M	46	ADC	T2N0M0	IB	II	Lung
30	M	50	SCC	T2N0M0	IB	III	Lung
31	M	77	ADC	T2N0M0	IB	II	Lung
32	M	57	SCC	T2N0M0	IB	I	Lung
33	F	70	ADC	T2N0M0	IB	II	Lung
34	F	70	ADC	T2N0M0	IB	II	Lung
35	M	55	Papillary ADC	T2N0M0	IB	N/A	Lung
36	M	65	SCC	T2N1M0	IIB	III	Lung
37	M	56	SCC	T2N0M0	IB	II~III	Lung
38	M	67	SCC	T2N0M0	IB	I~II	Lung
39	M	78	SCC	T2N0M0	IB	II~III	Lung
40	M	56	Papillary ADC	T3N0M0	IIB	N/A	Lung

Staging of lung cancer patients was done according to the TNM classification of lung carcinoma (2009). Grade I = well-differentiated; Grade II = moderately-differentiated; Grade III = poorly-differentiated. N/A = information not available.
