# Supplementary material for: Identification of beta-arrestin-1 as a diagnostic biomarker in lung cancer
Source: Br J Cancer. 2018 Aug 6;119(5):580–90. doi: 10.1038/s41416-018-0200-0 (PMC6162208; doi:10.1038/s41416-018-0200-0)
Supplement: Supplementary file 4 — Supp table 4 - Clinicopathological features of selected lung cancer patients from the US Biomax cohort, as provided by the manufacturer [file 41416_2018_200_MOESM4_ESM.pdf]

Supplementary Table 4. Clinicopathological features of selected lung cancer patients from the US Biomax cohort, as provided by the manufacturer.

**Features**		**Lung cancer diagnosis**	
		ADC	SCC
N° of subjects		27	44
Gender	Female	15	3
	Male	12	41
Age (y) [median value (range)]		57 (33-71)	61 (30-77)
Stage	I	7	16
	II	8	6
	III	12	22
Grade	I	5	1
	II	8	30
	III	13	13
	not available	1	0

Staging of lung cancer patients was done according to the TNM classification of lung carcinoma (2009). Grade I = well-differentiated; Grade II = moderately-differentiated; Grade III = poorly-differentiated.
