# Supplementary material for: Identification of beta-arrestin-1 as a diagnostic biomarker in lung cancer
Source: Br J Cancer. 2018 Aug 6;119(5):580–90. doi: 10.1038/s41416-018-0200-0 (PMC6162208; doi:10.1038/s41416-018-0200-0)
Supplement: Supplementary file 5 — Supp table 5 - Positivity of IHC staining in primary lung tumours in the in-house TMA according to tumour grade [file 41416_2018_200_MOESM5_ESM.pdf]

Supplementary Table 5. Positivity of IHC staining in primary lung tumors in the *in-house* TMA according to tumour grade.

IHC markers / Tumour grade	ARRB1-2		ARRB1		TTF1		NAPSA		KRT7		KRT5-6		p63	
	ADC	SCC	ADC	SCC	ADC	SCC	ADC	SCC	ADC	SCC	ADC	SCC	ADC	SCC
I	8/8	0/9	8/8	1/9	7/8	0/9	6/7	0/9	7/7	2/9	0/8	8/9	0/8	8/9
II	4/4	NA	5/5	NA	4/5	NA	3/4	NA	5/5	NA	0/5	NA	0/5	NA
II~III	1/1	NA	1/1	NA	0/1	NA	0/1	NA	1/1	NA	0/1	NA	0/1	NA
III	4/4	0/2	4/4	0/2	3/4	0/2	2/4	0/2	4/4	0/2	0/4	2/2	0/4	2/2

Data are shown as number of cases with positive staining/number of total cases in each category. Grade I = well-differentiated; Grade II = moderately-differentiated; Grade III = poorly-differentiated.
