# Supplementary material for: Identification of beta-arrestin-1 as a diagnostic biomarker in lung cancer
Source: Br J Cancer. 2018 Aug 6;119(5):580–90. doi: 10.1038/s41416-018-0200-0 (PMC6162208; doi:10.1038/s41416-018-0200-0)
Supplement: Supplementary file 6 — Supp table 6 - Positivity of IHC staining in primary lung tumours in the amsbio TMA according to tumour grade [file 41416_2018_200_MOESM6_ESM.pdf]

Supplementary Table 6. Positivity of IHC staining in primary lung tumors in the amsbio TMA according to tumour grade.

IHC markers / Tumour grade	ARRB1-2		ARRB1		TTF1		NAPSA		KRT7		KRT5-6		p63	
	**ADC**	**SCC**	**ADC**	**SCC**	**ADC**	**SCC**	**ADC**	**SCC**	**ADC**	**SCC**	**ADC**	**SCC**	**ADC**	**SCC**
**I**	N/A	0/3	N/A	0/3	N/A	0/3	N/A	0/3	N/A	0/3	N/A	3/3	N/A	3/3
**I~II**	1/1	0/4	1/1	0/4	0/1	0/4	1/1	0/4	1/1	0/4	0/1	4/4	0/1	4/4
**II**	8/8	0/1	8/8	0/1	7/8	0/1	7/7	0/1	8/8	0/1	0/8	1/1	0/8	1/1
**II~III**	2/2	0/4	2/2	0/4	2/2	0/4	2/2	0/4	2/2	0/4	0/2	4/4	0/2	4/4
**III**	2/2	4/9	1/2	4/9	1/2	0/9	1/2	0/9	1/2	2/9	0/2	7/9	0/2	7/9

Data are shown as number of cases with positive staining/number of total cases in each category. Grade I = well-differentiated; Grade II = moderately-differentiated; Grade III = poorly-differentiated. N/A = information not available.
