# Supplementary material for: Identification of beta-arrestin-1 as a diagnostic biomarker in lung cancer
Source: Br J Cancer. 2018 Aug 6;119(5):580–90. doi: 10.1038/s41416-018-0200-0 (PMC6162208; doi:10.1038/s41416-018-0200-0)
Supplement: Supplementary file 7 — Supp figure 1 - Beta-arrestin-1 (ARRB1) protein expression in lung ADC and SCC tissues and their distant « normal » counterparts [file 41416_2018_200_MOESM7_ESM.pdf]

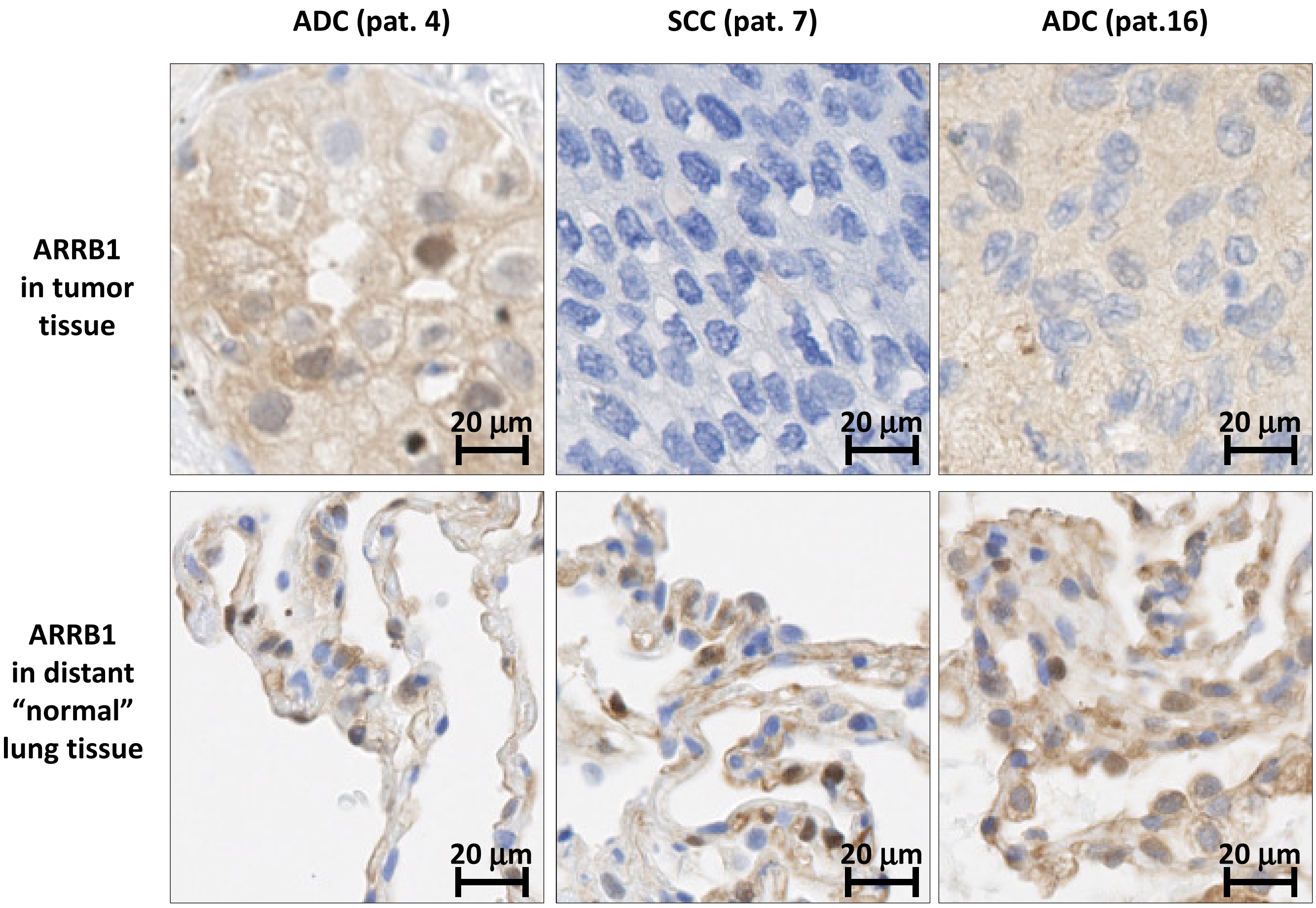

Supplementary Figure 1. Beta-arrestin-1 (ARRB1) protein expression in lung ADC and SCC tissues and their distant « normal » counterparts. Beta-arrestin-1 expression was assessed on sections from the *in-house* TMA by means of automated IHC using the BOND RX staining system and the DAB-based visualization Bond Polymer Refine detection kit (Leica Biosystems). IHC was performed using the anti-ARRB1 antibody (#30036) from Cell signaling. Results obtained in samples from 2 ADC and one SCC patients are shown.
