# Supplementary material for: Identification of beta-arrestin-1 as a diagnostic biomarker in lung cancer
Source: Br J Cancer. 2018 Aug 6;119(5):580–90. doi: 10.1038/s41416-018-0200-0 (PMC6162208; doi:10.1038/s41416-018-0200-0)
Supplement: Supplementary file 8 — Supp figure 2 - Expression of ARRB1 and ARRB2 transcripts in lung ADC, lung SCC and non-tumour samples [file 41416_2018_200_MOESM8_ESM.pdf]

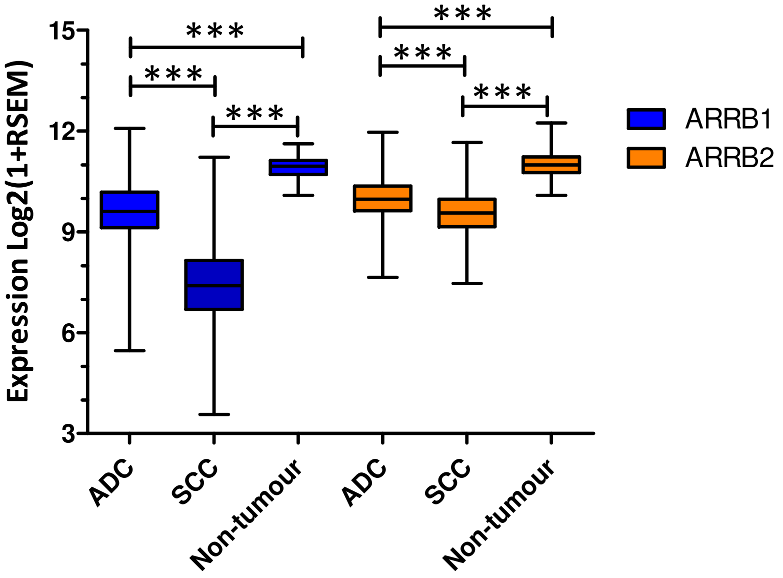

Supplementary Figure 2. Expression of *ARRB1* and *ARRB2* transcripts in lung ADC, lung SCC and non-tumour samples. Analysis of RNAseq (ILLUMINAHiSeq) datasets downloaded from TCGA Data Portal. Whiskers delimit the minimal and maximal values. n=515 ADC; n= 502 SCC and n= 110 non-tumoral lung tissue. Statistical analysis by one-way ANOVA with Tukey post-hoc analysis *** P ≤ 0.001 .
