# Supplementary material for: Identification of beta-arrestin-1 as a diagnostic biomarker in lung cancer
Source: Br J Cancer. 2018 Aug 6;119(5):580–90. doi: 10.1038/s41416-018-0200-0 (PMC6162208; doi:10.1038/s41416-018-0200-0)
Supplement: Supplementary file 9 — Supp figure 3 - Representative images of HPS and IHC stainings in the metastatic sites of lung ADC [file 41416_2018_200_MOESM9_ESM.pdf]

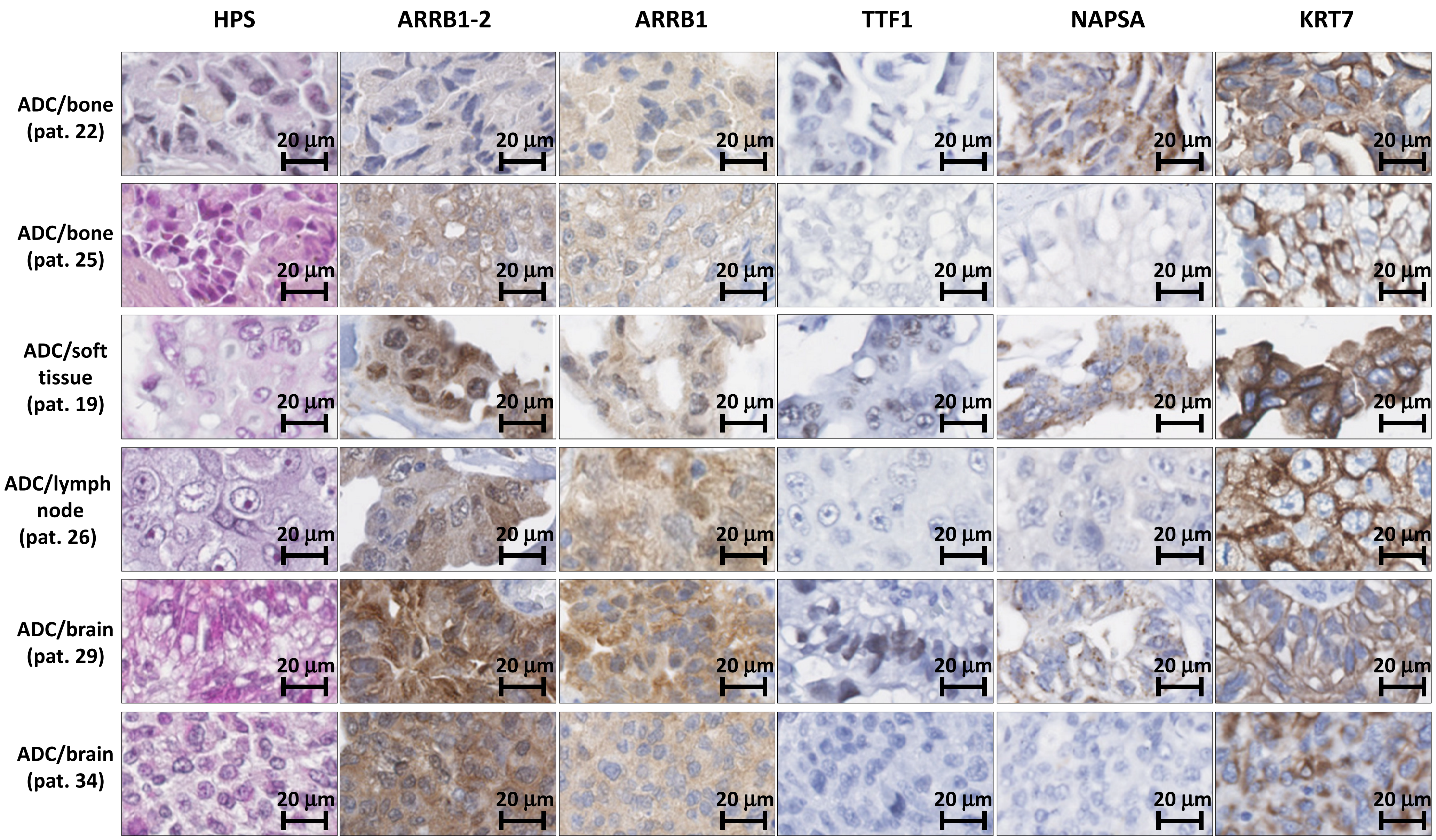

Supplementary Figure 3. Representative images of HPS and IHC stainings in the metastatic sites of lung ADC. Automated IHC was performed on sections from the *in-house* TMA using antibodies that recognize ARRB1-2, ARRB1, and the commonly used ADC markers TTF1, NAPSA and KRT7. DAB-based visualization was used to assess protein expression. For each sample, a Hematoxylin/Phloxine/Saffron (HPS)-stained slide is shown.
