# Supplementary material for: Identification of beta-arrestin-1 as a diagnostic biomarker in lung cancer
Source: Br J Cancer. 2018 Aug 6;119(5):580–90. doi: 10.1038/s41416-018-0200-0 (PMC6162208; doi:10.1038/s41416-018-0200-0)
Supplement: Supplementary file 10 — Supp figure 4 - Beta-arrestin-1-2 (ARRB1-2) protein expression in lung ADC and SCC samples in the US Biomax TMA [file 41416_2018_200_MOESM10_ESM.pdf]

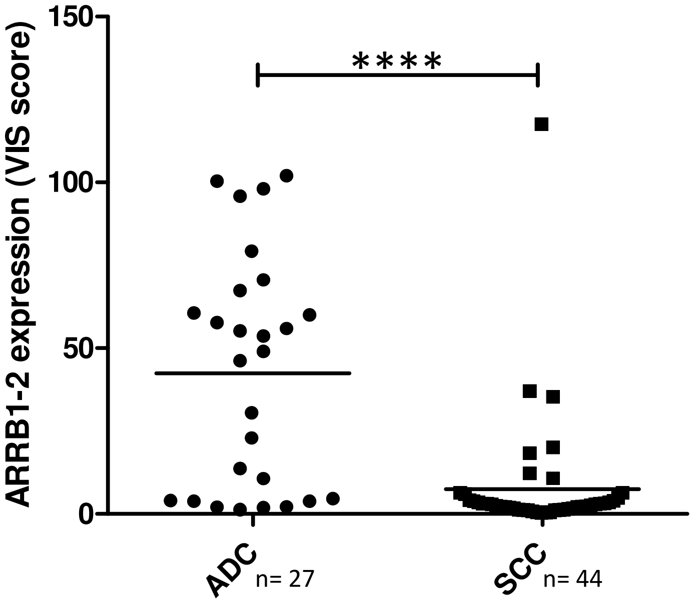

Supplementary Figure 4. Beta-arrestin-1-2 (ARRB1-2) protein expression in lung ADC and SCC samples in the US Biomax TMA. Scatter plots represent quantitative analysis (VIS score) of IHC results in a section of the US Biomax TMA incubated with an antibody that recognises both beta-arrestin-1 and 2 (beta-arrestin-1-2). “n” indicates the number of subjects in each group. Data points and their median are shown. **** P < 0.0001 using Mann-Whitney Rank Sum test.
