# Supplementary material for: Identification of beta-arrestin-1 as a diagnostic biomarker in lung cancer
Source: Br J Cancer. 2018 Aug 6;119(5):580–90. doi: 10.1038/s41416-018-0200-0 (PMC6162208; doi:10.1038/s41416-018-0200-0)
Supplement: Supplementary file 11 — Supp figure 5 - Baseline plasma levels of beta-arrestin-1 (ARRB1) protein in lung cancer patients who subsequently experienced different tumour responses to chemotherapy and EGFR inhibitors [file 41416_2018_200_MOESM11_ESM.pdf]

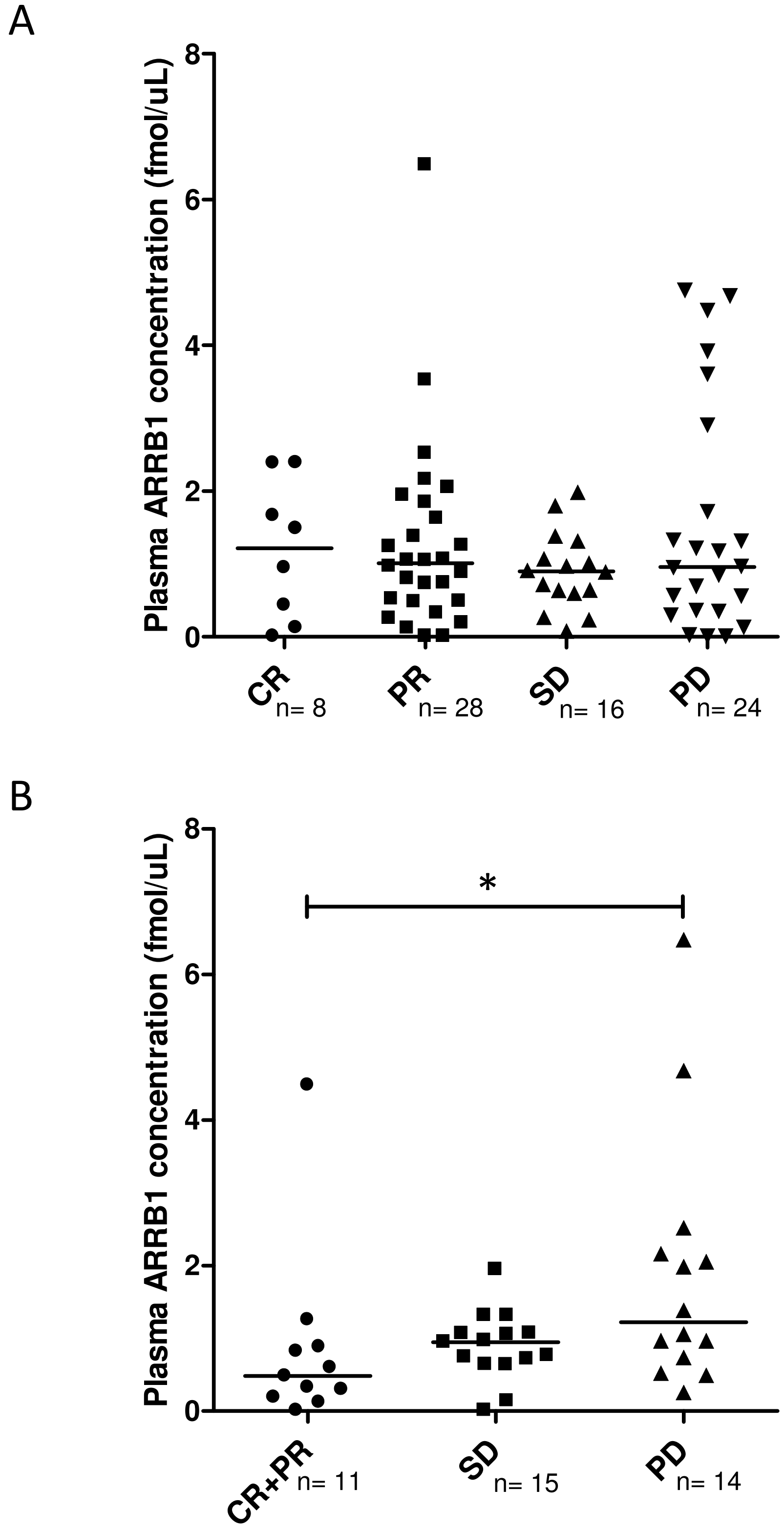

Supplementary Figure 5. Baseline plasma levels of beta-arrestin-1 (ARRB1) protein in lung cancer patients who subsequently experienced different tumour responses to chemotherapy and EGFR inhibitors. The PRM assay targeting EDLDVLGLTFR was used to quantify ARRB1 in plasma from lung cancer patients at time of sample collection, i.e. before treatment. Scatter plots represent baseline ARRB1 concentration in patients with different tumour responses following (**A**) classical chemotherapy and (**B**) EGFR inhibitor-therapy. The best response to therapy is reported for each patient. CR= complete response; PR= partial response; SD= stable disease and PD= progressive disease. "n" indicates the number of subjects in each group. Data points and their median are shown. * $P < 0.05$ using Mann-Whitney Rank Sum test.
